# Persistent metabolic changes in HIV-infected patients during the first year of combination antiretroviral therapy

**DOI:** 10.1038/s41598-018-35271-0

**Published:** 2018-11-16

**Authors:** N. Chantal Peltenburg, Johannes C. Schoeman, Jun Hou, Fernando Mora, Amy C. Harms, Selwyn H. Lowe, Jörgen Bierau, Jaap A. Bakker, Annelies Verbon, Thomas Hankemeier, Andre Boonstra

**Affiliations:** 1 Department of Internal Medicine, Division Infectious Diseases, Erasmus Medical Center, Wytemaweg 80, 3015 CE Rotterdam, The Netherlands; 2 Faculty of Science, Leiden Academic Centre for Drug Research, Analytical BioSciences, Einsteinweg 55, 2333 CC Leiden, The Netherlands; 3 Department of Gastroenterology and Hepatology, Erasmus Medical Center, Wytemaweg 80, 3015 CE Rotterdam, The Netherlands; 4 Department of Internal Medicine, Division Infectious Diseases, Maastricht University Medical Center, P. Debyelaan 25, 6229 HX Maastricht, The Netherlands; 5 Department of Medical Microbiology, School of CAPHRI, Maastricht University Medical Center, P. Debyelaan 25, 6229 HX Maastricht, The Netherlands; 6 Department of Clinical Genetics, Maastricht University Medical Center, P. Debyelaan 25, 6229 HX Maastricht, The Netherlands; 7 Department of Clinical Chemistry and Laboratory Medicine, Leiden University Medical Center, Albinusdreef 2, 2333 ZA Leiden, The Netherlands; 8 Department of Medical Microbiology and Infectious Diseases, Erasmus Medical Center, Wytemaweg 80, 3015 CE Rotterdam, The Netherlands

## Abstract

The HIV-human metabolic relationship is a complex interaction convoluted even more by antiretroviral therapy (cART) and comorbidities. The ability of cART to undo the HIV induced metabolic dysregulation is unclear and under-investigated. Using targeted metabolomics and multiplex immune biomarker analysis, we characterized plasma samples obtained from 18 untreated HIV-1-infected adult patients and compared these to a non-HIV infected (n = 23) control population. The biogenic amine perturbations during an untreated HIV infection implicated altered tryptophan- nitrogen- and muscle metabolism. Furthermore, the lipid profiles of untreated patients were also significantly altered compared to controls. In untreated HIV infection, the sphingomyelins and phospholipids correlated negatively to markers of infection IP-10 and sIL-2R whereas a strong association was found between triglycerides and MCP-1. In a second cohort, we characterized plasma samples obtained from 28 HIV-1-infected adult patients before and 12 months after the start of cART, to investigate the immune-metabolic changes associated with cART. The identified altered immune-metabolic pathways of an untreated HIV infection showed minimal change after 12 months of cART. In conclusion, 12 months of cART impacts only mildly on the metabolic dysregulation underlying an untreated HIV infection and provide insights into the comorbidities present in virally suppressed HIV patients.

## Introduction

The interaction of the Human immunodeficiency virus (HIV) with its host is a complex process with a growing body of literature revealing the capacity of HIV to induce a plethora of metabolic changes in the human body^1–5^. Hypertriglyceridemia was identified as one of the first metabolic consequences of HIV infection^6^. Since then, studies using Mass Spectrometry (MS) and Nuclear Magnetic Resonance (NMR) applied to biofluids of HIV-infected individuals have confirmed the presence of HIV induced metabolic alterations^1,7–9^. Investigating *in vitro* models of HIV infection using primary macrophages and CD4^+^ T-cells had different metabolic outcomes during HIV infection. CD4^+^ T-cells infected with HIV exhibited increased glucose uptake and upregulated glycolytic intermediates compared to reduced glucose uptake and steady-state glycolytic intermediates in HIV-infected macrophages^10^. While, *in vivo* models using rhesus macaques infected with simian immunodeficiency virus (SIV) revealed increased fatty acids, phospholipids, and acyl-carnitines, suggesting an impaired mitochondrial fatty acid oxidation^11^. Comparatively, serum and plasma derived from HIV infected individuals revealed altered metabolites of lipid and mitochondrial pathways as well as organic acids and fatty acids^1,5^. Moreover, the saliva from HIV-infected patients versus healthy controls revealed alterations in carbohydrate biosynthesis and degradation^2^.

Infection with HIV causes a progressive malfunctioning of the human immune system. HIV infection depletes CD4^+^ T-cells, while also inducing functionally exhausted CD8^+^ T-cells and impaired NK cells, leaving the host vulnerable to opportunistic infections^12^. Furthermore, the metabolic and immunological changes in untreated HIV patients increase the risk of developing comorbidities, including cardiovascular disease (CVD), insulin resistance and HIV-associated neurocognitive disorders (HAND)^13,14^.

The advent of combination antiretroviral therapy (cART) provided an important lifeline for HIV patients, since cART effectively inhibits HIV replication to undetectable levels, while also enabling the restoration of the immune system with increasing CD4^+^ T-cell counts. Nevertheless, important metabolic-related side-effects of cART are reported in patient populations, mainly lipodystrophy and insulin resistance, which may further predispose virally suppressed patients to increased CVD, diabetes, and kidney damage^15–17^. Also, studies have shown that although cART dramatically decreased the incidence of the most severe clinical phenotype of HAND (HIV associated neurocognitive disorder) – HIV-associated dementia - in virally suppressed patients the milder forms of HAND - asymptomatic neurocognitive impairment and mild neurocognitive disorders- have become more prevalent^18,19^. One aspect of the pathogenesis of CVD or HAND in successfully treated HIV patients is that while cART effectively suppresses HIV replication and activity, low levels of immune activation are sustained^20–22^. The ability of cART to rectify the HIV-induced metabolic dysregulation is unclear and under-investigated. However, this may be highly relevant since persistent metabolic stress could be an underlying pathogenic mechanism in the comorbidities of untreated HIV patients as well as cART suppressed HIV patients. Robust characterization of the metabolic alterations experienced during HIV infection is needed to determine the effect of cART on these pathways, in cART suppressed HIV patients.

To study this, we used comprehensive targeted metabolomics techniques integrated with classical immunological assays and compared changes in plasma of untreated HIV-1 patients to non-HIV infected individuals, and paired plasma of untreated HIV-1 patients at baseline to their plasma after 12 months of cART in the HIV-suppressed state, to assess whether profiles normalized to the situation in healthy individuals or remained perturbed. We found dysregulated biogenic amine and lipid metabolism in untreated HIV-infected patients, conditions that have been independently associated with the pathophysiology of CVD and HAND. After 12 months of cART, metabolic changes were found for some biogenic amines, while the lipid metabolites revealed increasing levels in the virally suppressed patients.

## Results

### Untreated HIV versus non-HIV-infected controls

#### Patient population A

Most of the individuals in both the patient and the control populations listed in Table 1 were male and Caucasians. Mean age did not differ between both groups. The mean CD4^+^ T-cell count of the HIV-1 patients was 441 × 10^6^ cells/L with an interquartile range (IQR) of 367.5 × 10^6^. The untreated HIV patients had a large heterogeneity in viral load with a median of 1.11 × 10^5^ viral copies and an IQR of 2.64 × 10^5^ especially when taking the minimum and maximum values as listed in Table 1 into account.

**Table 1:** Characteristics of the untreated HIV-infected patients (Population A) and the non-HIV infected control population.

Characteristics	Patients (n = 18)	Controls (n = 23)	P-value
Age; mean (±SD; years)	40 (10)	36 (10)	0.18
Male Gender (n/%)	16 (88.9%)	14 (60.9%)	0.08
Caucasian (n/%)	14 (77.8)	17 (73.9%)	0.90
CD4^+^ T-cell count; mean (±SD; x10^6^/L)	441 (265)	—	—
HIV viral load; median (min-max; copies/mL)	1.1 × 10^5^ (60–1 × 10^7^)	—	—

#### Altered plasma metabolic profiles in untreated HIV-infected patients

**Figure 1:**
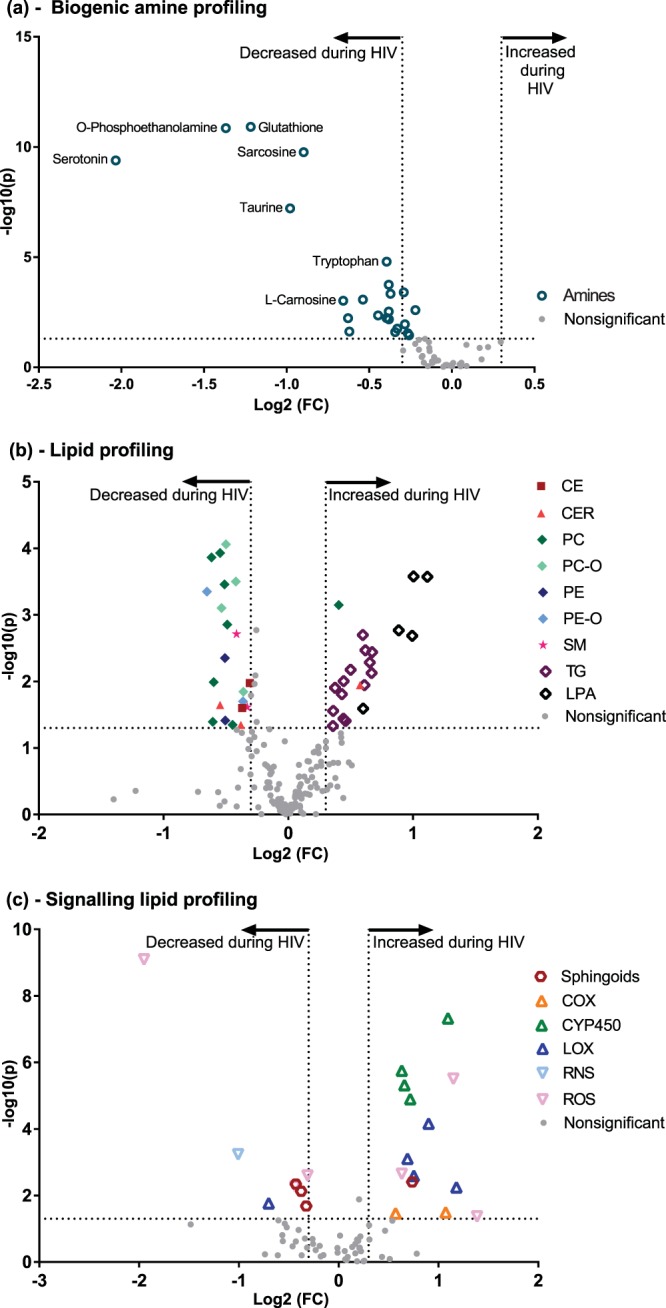
Plasma metabolic characterization of untreated HIV *vs* controls. Volcano plot of (**a**) the biogenic amine profile. (**b**) The lipid profile. (**c**) The signalling lipid profile. Volcano plots are representative of the −log10(Mann-Whitney p-value) on the y-axis with the x-axis showing the log2(Fold change) of the metabolite, between the controls and the untreated HIV patients (population A) respectively. Dashed lines represent the respective significance thresholds, with the significant metabolites identifiable by coloured symbols with either a name or corresponding class colour identifier. CE – Cholesterol esters; CER – Ceramides; PC – Phosphatidylcholines; PC-O - Plasmalogen PCs; PE – Phosphatidylethanolamines; PE-O – Plasmalogens PEs; SM - Sphingomyelins; TG - Triglycerides; LPA – Lysophosphatidic acid; COX - Cyclooxygenase; CYP450 – Cytochrome P450; LOX - Lipoxygenase; RNS – Reactive nitrogen species; ROS – Reactive oxygen species.

To characterized metabolic alterations experienced during an untreated HIV-1 infection, we compared 18 untreated HIV-1-infected patients (population A) with 23 controls through profiling their biogenic amines, lipids, and signalling lipid metabolites. As shown in the volcano plot in Fig. 1a, 18 biogenic amines were differently affected in HIV patients versus controls, with reduced levels in all (see Supplementary Table S1). Reduced levels of antioxidants, including total glutathione and taurine, together with lipid headgroup moieties o-phosphoethanolamine and the choline metabolite sarcosine were identified as the most significantly altered amine metabolites during an untreated HIV infection. Furthermore, untreated HIV patients had decreased levels of tryptophan and serotonin compared to controls. The plasma kynurenine/tryptophan (K/T) ratio, as a readout for indoleamine 2,3-dioxygenase (IDO) activity, showed upregulated IDO activity during untreated HIV infection compared to controls (p = 0.0003) (see Supplementary Fig. S1A). Concurrently investigating the plasma serotonin/tryptophan ratio reflected the consequences of increased IDO activity for serotonin synthesis during untreated HIV infection compared to controls (p < 0.0001) (see Supplementary Fig. S1B). Decreased levels of the branch chain amino acids leucine and isoleucine as well as carnosine reflected impaired muscle metabolism during an untreated HIV infection. The significantly decreased levels of ornithine and putrescine in untreated HIV patients also hints at a reduced urea cycle and thus ammonia clearance in these patients compared to controls. Additionally, decreased levels of alanine, asparagine, α-aminobutyric acid, tyrosine, and methionine were also detected in untreated HIV patients compared to controls. Evaluation of the levels of plasma lipids, which included the different lysophospholipids classes, phospholipids, di/triglycerides, sphingomyelins, ceramides, and cholesterol esters, revealed 44 modulated metabolites with a p < 0.05 and a fold-change (FC) ≥ 1.30 or ≤0.70 (Fig. 1b and Supplementary Table S2). The lysophospholipids class presented with 6 lysophosphatidic acid (LPA) species having increased plasma levels in untreated HIV-infected patients versus controls, and one lysophosphatidylcholine (LPC) metabolite which was reduced compared to controls. Furthermore, two monounsaturated sphingomyelin (SM) species, two PUFA cholesterol ester (CE), and two long-chain saturated ceramides (CER) metabolites all had reduced levels in untreated HIV patients. The triglycerides (TG) profile presented with 14 mostly polyunsaturated triglycerides species showing significantly increased levels during untreated HIV infection. The phospholipids presented with seven phosphatidylcholines (PC), four plasmalogen phosphatidylcholines (PC-O), two phosphatidylethanolamines (PE) and two plasmalogen phosphatidylethanolamines (PE-O) all having decreased levels during untreated HIV.

The third subclass of metabolites that were evaluated for differences between untreated HIV patients and controls was the class of signalling lipid mediators, derived from the enzymatic or free radical oxidised polyunsaturated fatty acids, which includes the prostaglandins, thromboxanes, hydroxy-fatty acids, leukotrienes, resolvins, epoxy-fatty acids, isoprostanes and nitro fatty acids. During the untreated HIV infection 22 significantly affected signalling lipid metabolites were identified of which 8 had reduced levels and 14 had increased levels in the untreated HIV patients compared to controls. (Fig. 1c, Supplementary Table S3). The four signalling mediators: 5,6-DiHETrE, 8,9-DiHETrE, 11,12-DiHETrE, and 14,15-DiHETrE all derived from arachidonic acid and synthesised by cytochrome P450 isozymes showed the most significant increased levels in the untreated HIV patients compared to the controls. Furthermore, untreated HIV patients had increased levels of dihydrosphingosine but decreased levels of three different Sphingosine-1-phosphate species compared to controls. The metabolites 5-HETE, 12-HETE and 15-HETE derived from the Lipoxygenase activity on Arachidonic acid, also had increased levels compared to the controls. Oxidized lipids due to the activity of ROS and RNS showed differential responses with increased levels of 8-iso-PGE2, 2,3-dinor-PGF2a and 20 HETE and decreased levels of 11-HDoHE and 8,12-iPF2α-IV in untreated HIV patients compared to controls.

### Untreated HIV versus HIV-suppressed (12 months of cART)

#### Patient population B

The characteristics of the HIV-1-infected patients selected to compare baseline metabolic profile to the HIV-suppressed profile after 12 months of therapy (population B) are presented in Table 2. This is an independent population from the untreated HIV-infected population A introduced in Table 1. Most of the patients had a male gender. Mean CD4^+^ T-cell count at the start of cART was of 267 × 10^6^ cells/L with an IQR of 224 × 10^6^. There was a large heterogenicity in viral load with a median of 5.5 × 10^4^ copies/ml and an IQR of 2.7 × 10^5^. 89% of the patients were of European descent. Four patients had a blip in their viral load at 12 months on cART: their viral loads were 53, 73, 103 and 130 copies/ml. Most patients used Abacavir in combination with Lamivudine as the backbone in cART (n = 25). The patients who only used NRTI’s all used the combination Abacavir with Lamivudine and Zidovudine (n = 8) except for one patient who used Abacavir/Lamivudine with Stavudine. The protease inhibitors (PI) used were Lopinavir (in combination with ritonavir) (n = 4), Atazanavir (n = 1) and Darunavir (n = 1). The NNRTI’s used were Efavirenz (n = 8), Nevirapine (n = 4) and Rilpivirine (n = 1). Five patients used a statin. The incidence of dyslipidaemia or the use of lipid-lowering therapy (statins and/or fibrates) remained stable during the first year of cART.

**Table 2:** Characteristics of 28 HIV-infected patients (Population B) at baseline and after 12 months of cART.

Characteristics	At baseline^a^	±12 months of cART
Age; mean (±SD; years)	40 (10)	
Male Gender (n/%)	21 (75.0%)	
European region of origin (n/%)	25 (89.3%)	
Statin use (n/%)	4 (14.3%)	5 (17.9%)
Mean Cholesterol^b^ > 6.5^c^ (n/%)	unknown	6 (21.4%)
Mean LDL > 4.5^c^ (n/%)	unknown	4 (14.2%)
Mean Triglycerides > 2.3^c^ (n/%)	unknown	8 (28.6%)
BMI^d^; mean (±SD; kg/m^2^)	22.7 (3.1)	23.1 (3.3)
Hepatitis B coinfection (n/%)	1 (3.6%)	
Hepatitis C coinfection (n/%)	8 (28.6%)	
Alcohol ≥ 5 IE/day (n/%)	1 (3.6%)	
Hard drugs^e^ or methadone use (n/%)	8 (28.6%)	
CD4^+^ T-cell count^f^ mean (±SD; x10^6^/L)	267.4 (158.9)	489.0 (209.4)
Time on cART; mean (±SD; months)	n.a.	12.5 (1.2)
HIV viral load < 10^2^ ^g^ (n/%)	0 (0%)	26 (93.6%)
HIV viral load > 10^2^ ^g^ (n/%)	28 (100%)	2 (7.2%)
HIV viral load^g^; median (min-max)	5.5 × 10^4^ (1.6 × 10^3^ – 8.1 × 10^5^)	
cART regimen NRTI^h^ + NNRTI^i^ (n/%)	n.a.	13 (46.4%)^k^
cART regimen NRTI^h^ + PI^j^ (n/%)	n.a.	6 (21.4%)^k^
cART regimen NRTI^h^ only (n/%)	n.a.	9 (32.1%)^k^

^a^cART = combination Anti-retroviral therapy; ^b^mean value of 6 months prior to sample used; ^c^mmol/L; ^d^BMI = Body mass index; ^e^cocaine, heroin and/or MDMA (3,4-methylenedioxymethamphetamine); ^f^x10^6^/L; ^g^copies/ml; ^h^NRTI = Nucleoside reverse transcriptase inhibitor; ^i^NNRTI = Non-nucleoside reverse transcriptase ^j^PI = Protease inhibitor; ^k^Three patients used a different cART regimen at 6 months versus 12 months of cART. Because of one patient switching from a protease inhibitor (PI) based regimen to a non-nucleoside reverse transcriptase inhibitor (NNRTI) or nucleoside reverse transcriptase inhibitor (NRTI) only regimen and vice versa, the proportion of PI-based, NNRTI based or NRTI the only regimen remained unchanged in the entire group.

#### Metabolic consequences following cART treatment of HIV patients

**Figure 2:**
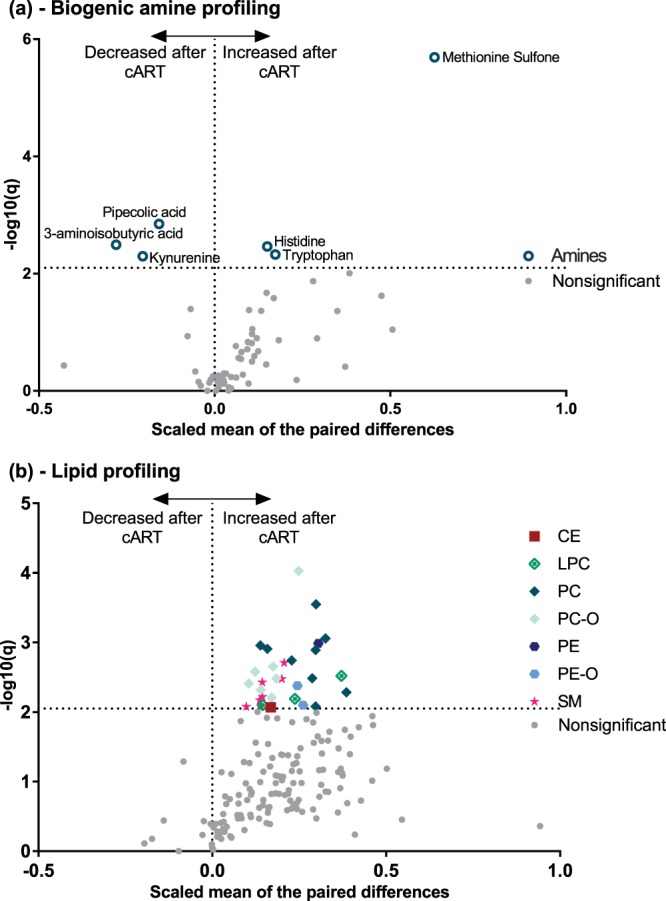
Plasma metabolic characterization of response to 12 months’ cART. Modified volcano plots showing (**a**) the biogenic amine profile, and (**b**) the lipid profile. The modified volcano plots are representative of the −log10(FDR adjusted paired t-test p-value) on the y-axis with the x-axis showing the scaled mean of the paired differences per metabolite, between the baseline and 12 months cART follow-up sample (patient population B). The horizontal dashed line represents a false discovery rate adjusted q-value < 0.05. Significant metabolites are identifiable by coloured symbols with either a name or corresponding class colour identifier. CE – Cholesterol esters; LPC – Lysophosphatidylcholines; PC – Phosphatidylcholines; PC-O - Plasmalogen PCs; PE – Phosphatidylethanolamines; PE-O – Plasmalogens PEs; SM – Sphingomyelins.

Next, we evaluated the metabolic changes associated with 12 months of cART treatment, as well as HIV suppression in the longitudinal patient population B. cART treatment of the HIV patients resulted in viral suppression with undetectable plasma HIV RNA levels and partial restoration of CD4^+^ T-cell counts in the blood (Table 2). Eight HIV infected patients were HCV positive, but were untreated for HCV during the 12-month period and were not excluded from the analyses. The untreated HCV infection had minimal impact on the metabolic findings as can be seen in see Supplementary Fig. S2. Evaluation of biogenic amine levels in paired plasma samples showed significantly increased methionine sulfone, histidine and tryptophan levels during the therapy period as compared to the baseline samples (untreated HIV), whereas pipecolic acid, 3-aminoisobutyric acid, and kynurenine showed significantly decreased levels during the therapy period as compared to the baseline (Fig. 2a, Supplementary Table S4). Significant metabolites were identified by having a false discovery rate adjusted p-value < 0.05 using a paired t-test approach. The K/T ratio revealed significantly decreased IDO activation after 12 months of cART (see Supplementary Fig. S3), however, the ratio between tryptophan and serotonin showed no significant changes. The neurotransmitter dopamine and metabolite α-aminobutyric acid correlated with CD4^+^ T-cell counts during 12 months of therapy (see Supplementary Fig. S4A).

The lipid profile showed an overall increasing trend reflecting higher levels after the 12-month cART period compared to their baseline levels, as can be seen in Fig. 2b. Predominantly it was the phosphatidylcholine metabolism responding to cART with significantly increased levels of 9 phosphatidylcholine species, 8 plasmalogen phosphatidylcholine, and 3 lysophosphatidylcholine species after 12 months of cART compared to baseline values prior to starting of cART. Furthermore, 7 sphingomyelin metabolites, together with one phosphatidylethanolamine species, two plasmalogen phosphatidylethanolamine, and a cholesterol ester were also increased after 12 months of cART compared to their baseline values. Furthermore, the phosphatidylcholines also revealed a positive association with CD4^+^ T-cell counts of the patients (see Supplementary Fig. S4B).

Finally, we compared changes in the signalling lipid profiles in patient plasma after 12 months of cART to their untreated HIV levels and found no significant changes.

### Plasma immune-metabolic networks of HIV infection and therapy

Plasma levels of cytokines and immune mediators are a commonly used readout reflecting systemic immune activity^23,24^. To correlate metabolite profiles in plasma with immune activity, we determined in subsequent plasma samples of the same patients the levels of the infection markers IP-10, sIL-2R, and D-dimer, the inflammation markers IL-6, IL-10, IL-21, IL-18, and MCP-1, and the soluble exhaustion markers sPD1, sPD-L1, sPD-L2, and sTIM3. Significantly enhanced plasma levels of D-Dimer, IL-18, MCP-1, sPD-L2, sTim-3, IP-10, sIL-2R, CRP, and sTNF-RII were observed in patients with untreated HIV as compared to non-HIV infected controls (Population A), and are shown in Supplementary Fig. S5. The other mediators tested were below the limit of detection of the assay, which included IL-6, IL-10, IL-21, sPD1, and sPD-L1.

Evaluating the changes in the immunological parameters in population B after the 12 months cART period revealed an immunological picture of effective therapy shown in Supplementary Fig. S6. Markers of infection revealed that after the initiation of cART, both plasma IP-10 and sIL-2R levels were reduced significantly. Furthermore, initiation of cART reduced the levels of the inflammation marker IL-18 whereas MCP-1 levels remained unchanged when comparing baseline to 12 months of cART. An improvement in markers of exhaustion was found with reducing levels of both sPD-L2 and sTIM3 after 12 months of cART.

**Figure 3:**
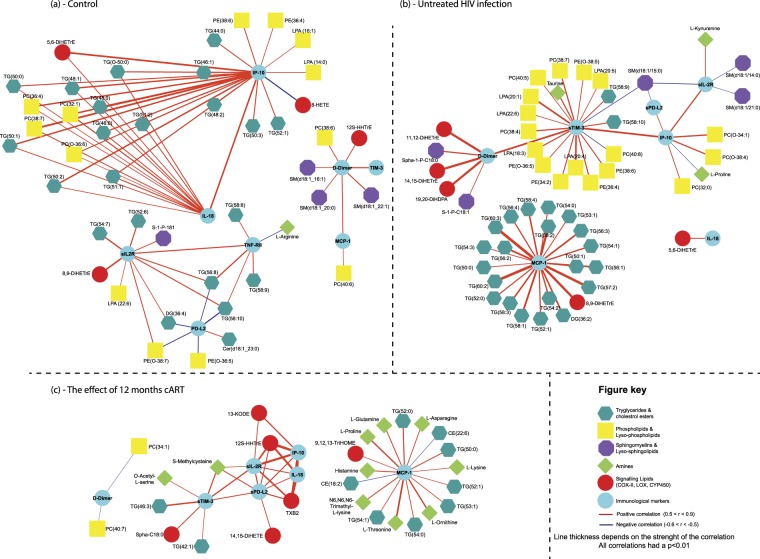
Immune-metabolic networks of controls, untreated HIV and the influence of cART. (**a**) Control immune-metabolic correlation network based on correlating the absolute levels of plasma metabolites and plasma cytokines in the control group. (**b**) Untreated HIV immune-metabolic correlation network based on correlating the absolute levels of plasma metabolites and plasma cytokines in the untreated HIV group (Population B). (**c**) The immune-metabolic correlation network following 12 months cART. Using the paired samples of population B, the relative change in levels of 12 months’ cART compared to their baseline levels (untreated HIV sample) were determined for plasma metabolites and cytokines and subsequently correlated. For all three networks, spearman correlations were performed and significant correlations were defined as having a strict p-value (two-tailed) < 0.01.

Next, we constructed immune-metabolic correlation networks respectively for the controls and the patients with an untreated HIV infection from population B using the absolute of levels of metabolites and immune mediators. Figure 3 presents these three immune-metabolic correlation networks showing all correlations with a strict p-value cut-off of p < 0.01. Comparing Fig. 3a (the control network) to Fig. 3b (the untreated HIV network) difference is seen in the interactions between the two networks. The control network revealed isolated clusters with IP-10 and IL-18 correlating to different lipid classes, sIL-2R and sTNF-RII correlated together and D-Dimer, sTIM3 and MCP-1 showing correlation. Comparatively the untreated HIV shows positive correlations between sTIM-3, sIL-2R, IP-10 and sPD-L2 with D-Dimer connecting the network through sTIM-3, revealing correlations between markers of infection and exhaustion. In both networks, sTIM-3 and D-Dimer revealed strong positive correlations, while in the untreated HIV patients sTIM-2 also showed strong interactions with phospholipids as well as lysophospholipids. Furthermore, in untreated HIV patients MCP-1 correlated strongly to several triglyceride species compared to the control network where MCP-1 correlate only to D-Dimer. Interestingly, in the control network, IL-18 showed strong interactions with triglyceride species, whereas IL-18 was correlated to only 5,6-DiHETrE in the untreated HIV patients.

Next, for the influence of cART on HIV infection, we constructed immune-metabolic correlation network based on the relative change in levels of metabolites and immune mediators in the paired plasma of the patients on 12 months cART versus their untreated HIV levels. As shown in Fig. 3c, the correlation network revealed distinct immune-metabolic interactions. Firstly, changes in sTIM-3, sIL-2R, IP-10, sPD-L2 and IL-18 positively correlated with each other, with D-dimer becoming disconnected from the core network. Again, MCP-1 showed minimal interactions to other immune mediators in the panel: changes in triglyceride levels correlated strongly to changes in MCP-1 levels as did some amine metabolites, while changes in two cholesterol-esters showed negative correlations. Furthermore, cART diminished the interaction of sTIM-3 with the phospholipids and lysophospholipids which were found in untreated HIV-patients.

## Discussion

The data presented in this study provide some insight into the distinct effects of HIV and cART have on metabolism. Through identifying immune-metabolic pathways altered during untreated HIV infection, we can follow these pathways during the first 12 months after commencing cART in the same patients, monitoring their responses. In line with earlier research, we found decreased glutathione, taurine and tryptophan levels as well as upregulated IDO activity in untreated HIV patients compared to HIV-seronegative controls^8,25,26^. Also, increased levels of highly unsaturated long-chain triglycerides^27^ and decreased levels of sphingomyelin species characterized an untreated HIV Infection compared to controls. After the initiation of cART, changes in the lipid metabolism were primarily found within the phospholipids while the highly unsaturated triglyceride species remained elevated. A noteworthy metabolic finding is the unresponsive signalling lipid profile, showing minimal changes after the first 12-month cART period. Signalling lipids including eicosanoids are metabolically derived immunological mediators and might represent a metabolic reflection of the immune competence of HIV-infected patients^28,29^. The unresponsive signalling lipid metabolism could result from the sequestering of their polyunsaturated fatty acid precursors into triglyceride species. Cassol *et al*. reported decreased eicosanoid levels specifically 5-hydroxyeicosatetraenoic acid (5-HETE), prostaglandin B2, prostaglandin E2 and thromboxane B2 in cART-treated HIV-positive individuals compared to controls^1^. Since eicosanoids also play an essential role in immunological crosstalk^30^ it could help to explain the dysregulated immune surveillance and exhausted phenotype persisting in virally suppressed patients^31^.

Studying the immune-metabolic networks revealed that the initiation of cART disconnected the interactions between kynurenine and sIL-2R as well as sTIM-3’s interactions with phospholipids and lysophospholipids. Thus, HIV suppression through cART attenuates the pro-inflammatory sIL-2R and IDO pathways differently, since it has been reported that IDO activity remains significantly increased compared to controls even after 24 months of cART^32^. Secondly, cART might contribute to an exhausted immune phenotype through dysregulating the phospholipids (increased during the first 12 months of cART) and their interactions with sTIM-3 interactions. Further, IL-18, a pro-inflammatory cytokine found elevated in advanced stages of HIV infection^33^, showed strong correlations to triglyceride levels in the control population. The role of IL-18 in cardiovascular disease and the metabolic syndrome, as well as its association with triglyceride levels, has been previously described^34,35^. Comparatively, in the untreated HIV infected population, the triglyceride species showed a strong correlation to MCP-1 levels and not to IL-18. Initiating 12 months of cART was unable to nullify this interaction, revealing that changes in triglyceride levels still strongly correlated to those of MCP-1 in the paired patient samples. Mihăilescu *et al*., found that the prevalence of insulin resistance and metabolic syndrome was higher in HIV suppressed patients compared to virally active patients, and that the levels of MCP-1 correlated to both these co-morbidities^36^, supporting our findings.

Disentangling the mechanism behind the observed metabolic changes during an HIV infection and therapy is a complex task. However, the importance of this task is stressed by the findings in this study that even 12 months of cART does not attenuate metabolic dysregulation. This is opposed to the finding that immune activation in HIV-infected patients is normalized during treatment with cART for this same period, as we also previously showed^33^. Sustained immune activation thus is not the complete answer to what causes co-morbidity in HIV-infected patients, like neurocognitive disorders and cardiovascular disease. Although cART has become increasingly effective over the last years, but as we show here, is not enough to fully restore the metabolic profile of HIV-suppressed patients to normal.

This finding warrants further research, to contribute to unravelling the metabolic pathophysiological pathways in HIV comorbidities, with multiple findings in this study able to generate testable hypothesis regarding HAND and CVD. With respect to HAND, the following questions arise applicable to both untreated HIV and cART-treated HIV patients. (1) How does the sustained elevated Indoleamine-2,3-dioxygenase (IDO) activity (the rate-limiting enzyme catabolizing L-tryptophan into neurotoxic metabolites and not serotonin) contribute to neurological disorders? (2) What is the pathological mechanism underlying a dysregulated urea cycle during an untreated HIV infection? Since disorders of the urea cycle are known to manifest with neurological implications due to hyperammonemia^37^. Similarly to HAND, questions relating to CVD, an important cause of morbidity and mortality in untreated and suppressed HIV-infected patients, arises^38,39^. (3) Do the significant correlations between changes in triglycerides and MCP-1 levels, expose an alternative mechanism contributing to the development of CVD, independent of IL-18? (4) How does an unresponsive eicosanoid metabolism contribute to CVD, since they play an important role in normal cardiovascular function?^40–42^. Potentially with the answer to these questions, morbidity, and mortality in HIV-infected patients because of the non-communicable disease can be further diminished.

It is important to note that in our study the time of follow-up of these patients was too short to draw conclusions about causality. The use of targeted metabolomics platforms in conjunction with other omics technologies would provide a unique opportunity to study HIV pathogenic mechanisms, as well as to identify biomarkers relevant to co-morbidities relating to both HIV and the use of ARVs. Studies investigating the metabolic dysregulation of HIV infections and exposure of antiretrovirals can be further strengthened by investigating paired longitudinal samples with a known outcome. Furthermore, these techniques could also proof complementary to antiretrovirals pharmacokinetic studies currently in clinical trials.

It is important to mention that due to the retrospective nature of the part of our study in which we compared cART naïve HIV-infected patients with their samples after 12 months of therapy, it is difficult to ascertain whether all blood samples were processed identically, thereby introducing some variation in outcome parameters. Further, the duration of the HIV infection prior to starting cART is not known of these patients. Apart from the HIV infection, other factors might play a role in metabolic changes, for instance, smoking habits, aging, and timing of sampling of the material. Also, we have no data on the fasting state of both the patient as well as the control population, however, because the samples of the included patients were taken during routine outpatient visits and blood donors, in general, are instructed not to donate blood in the fasted state, it is safe to assume that both groups were in a post-prandial state. Food intake can affect metabolites and phospholipid levels^43,44^, but also multiple large population studies have shown that changes in plasma lipids and lipoproteins change only modestly during the day, in response to habitual food intake^45,46^. Further, although sub-analysis of 12 months cART according to PI or NNRTI used did not show differences in lipid profiles, the choice of the specific components of the cART regimen might be a significant factor or influence on the metabolic profile of a patient in the long run. For instance, abacavir use, which is associated in the literature with an increase of CVD^47,48^. Population A and B differed significantly from each other regarding mean CD4+ T-cell counts and cytokine profiles, probably due to the fact that Population A was selected to be compared to a non-HIV infected, healthy control population and therefor selection criteria was more strictly defined than those for population B, where the patients were their own controls. In population B, five patients were using statins during the first year of cART, of which four were already receiving statin therapy prior to the start of cART. We were unable to identify any trends in the data indicating that lipid profiles were skewed based on the use of statins. Eight patients were coinfected with HCV, which may have had an effect on the comparison of the untreated patients compared to controls. However, during this study, none of the patients was treated for HCV, thus this was a stable factor in the comparison between untreated HIV and 12 months of cART. A sub-analysis leaving out the HCV coinfected patients showed minimal impact on the outcome. In this study, we were not able to include a sample of the control patients after one year. However, to determine the baseline stability of the metabolic/immune network, further studies are needed with follow-up samples of a control population.

In conclusion, in our study, we found significant changes in the metabolism relevant of untreated HIV infected patients and after 12 months cART. Additional to this finding, and potentially even more important is the finding that cART alone does not restore these changes. Further insight into the metabolic changes caused by HIV infection is warranted to optimize therapy in addition to cART for this patient population.

## Methods

### Patients and sample collection

#### Untreated HIV versus non-HIV-infected controls, population A

For the comparison of metabolic profiles of untreated HIV-1-infected patients to a non-HIV infected control population, 18 HIV-1-infected, cART naïve patients were selected from the outpatient clinic of the Erasmus Medical Center in Rotterdam, The Netherlands, archived sample bank. The plasma was stored at −80 °C. Inclusion criteria were age over 18 years and no previous treatment for HIV. Exclusion criteria were severe comorbidity (e.g. diabetes mellitus, cardiovascular disease, opportunistic infection), coinfection with hepatitis B or C, use of alcohol > 2 IU/day and use of co-medication. All patients had given written informed consent for inclusion in the database of the Dutch HIV monitoring foundation (Stichting HIV Monitoring; SHM) also known as the AIDS Therapy Evaluation in the Netherlands (ATHENA) cohort, and collection of demographic, laboratory and clinical data from the medical records and storage and future use for scientific research of biological material. Their plasma samples were compared to a non-HIV infected control population from volunteers (n = 23), without comorbidity or co-medication, which had all given their written informed consent.

#### Untreated HIV versus HIV-suppressed (12 months of cART), population B

For the comparison of metabolic profiles of untreated HIV-1-infected patients to the HIV-suppressed situation, plasma samples of 28 HIV-1-infected patients from the outpatient clinic of the Maastricht University Medical Center in Maastricht, The Netherlands were selected in the archived sample bank. The plasma was stored at −80 °C. All patients had given written informed consent for inclusion in the database of the Dutch HIV monitoring foundation and collection of demographic, laboratory and clinical data from the medical records and storage and future use for scientific research of biological material. From each included patient, a plasma sample was selected from before the start of cART and at 12 months after the start of cART. All patients were older than 18 years at the start of cART and had not received any previous ART therapy. All patients started with a cART regimen containing Abacavir. The patients had no diabetes mellitus and no diagnosed autoimmune diseases. We retrieved information regarding dyslipidaemia from the earlier laboratory results prior to the 12 months samples after the start of cART. Because the patients were generally not in care prior to the HIV diagnosis, no values were available from the blood sample prior to the start of cART. The study was performed according to the Helsinki Declaration and approved by the Ethical Committee of the Maastricht University Medical Center.

### Targeted LC-MS metabolomics

Targeted metabolomics analyses were performed using standard operating procedures derived from previously published methods^49–52^. Detailed procedures and target lists are provided in the Additional file 1 - Methods with a brief overview of the four platforms used given in Table 3. After LC-MS analyses, peak integration was done using the instrumental software, and the relative ratios between metabolites and their corresponding internal standards were determined.

**Table 3:** Metabolomics platforms. A brief overview of the platforms detailing volumes, sample preparation, and analytical instruments.

Targeted Metabolomics Platform	Volume serum used (μL)	Sample Prep Method	Analytical platform	Metabolite class coverage	Platform Targets (n)		
					Total	Quality control passed	% Missing data
Biogenic amine^50^	5 μL	Protein precipitation & AccQ-Tag derivatization	UPLC-TQMS	Amino acids, catecholamines & polyamines	100	62	0%
Positive lipid^49^	10 μL	Isopropyl alcohol extraction	UPLC-QToF	Lysophospholipids, phospholipids, cholesterol esters, di/triglycerides & sphingomyelins	250	147	0.02%
Oxylipins^51^	250 μL	Oasis HLB SPE extraction	HPLC-MS/MS	Hydroxylated fatty acids, prostaglandins & thromboxanes	120	68	7.7%
Oxidative stress^52^	150 μL	Butanol:Ethyl acetate liquid-liquid extraction	UHPLC-MS/MS Low pH run	Isoprostanes, nitro-fatty acids, sphingosine & sphinganine	46	36	6.6%
			UHPLC-MS/MS High pH run	Sphingosine-1-phosphate & lysophosphatidic acids species.			

UPLC – Ultra Pressure Liquid-Chromatography; TQMS – Triple quadrupole mass spectrometer; QToF – Quad Time of Flight; HPLC - High-Pressure Liquid-Chromatography; UHPLC – Ultra High-Pressure Liquid-Chromatography; MS/MS – Triple quadrupole Mass Spectrometer.

#### Metabolomics quality controls

Quality control (QC) samples consisted of equal aliquots of a QC pool made by combining equal volumes (±25 µL) of all study samples. A set of QC samples was then included during the analyses of the experimental groups on the individual metabolomic platforms and evenly distributed across the randomized samples prior to LC-MS analyses. In addition, independent duplicate samples (10–15%) were randomly selected. Using the QC samples and duplicate samples, a double-QC approach was applied to include metabolites that were reliably measured by the individual metabolomics platforms by reporting and using only those metabolites for which both duplicate samples and QC samples showed an RSD < 30%.

### Multiplex immunoassays to assess plasma biomarkers

The levels of cytokines, chemokines, growth factors and other proteins were determined using the Procarta Plex human Immune Monitoring Panel (Affymetrix, Vienna, Austria). The panel measured 14 proteins simultaneously and consisted of IP-10, IL-10, IL-6, D-dimer, sIL-2R, IL-21, IL-18, MCP-1, sPD-L1, sPD-L2, sTIM3, sPD1, CRP, and sTNF-RII. The assay was conducted according to the manufacturer’s instructions, identical to the procedures used in our previous studies^53–55^. The concentrations of analytes were measured using the microsphere-based multiplex Luminex-100 (Luminex Corporation, Austin, TX, USA), Samples to compare untreated HIV with non-HIV controls were analyzed in a different run than paired samples of untreated and HIV-suppressed patients. Data were analyzed using ProcartaPlexAnalyst 1.0 (www.ebioscience.com/resources/procartaplex-analyst-1.0-software.htm).

### Statistical analyses

SPSS 21.0 (SPSS Inc., Chicago, IL, USA) was used for Fisher’s exact tests on the patient cohort characteristics presented as frequencies, and ANOVA on the continuous values. A combination of univariate and multivariate bioinformatics approaches was performed using the R script‒based online tool Metaboanalyst 3.0, a comprehensive tool suitable for analysing metabolomics data^56^. The metabolomics datasets were log transformed and auto-scaled prior to bioinformatics analyses. For the analyses between controls and untreated HIV patients, significant metabolites were identified per metabolomics platform based on the following criteria: i. a p-value < 0.05 using the unpaired student t-test, and ii. a fold change (FC) ≥ 1.30 or ≤0.70, indicating a 30% increase or decrease. The False discovery rate’s q-values are reported for every reported p-value. For the analyses between the paired untreated HIV patients and 12 months cART follow up, significant metabolites were identified per metabolomics platform based on a False discovery rate adjusted p-value < 0.05 using a paired student t-test. Plasma protein biomarkers were analysed using the unpaired student t-test for comparing controls and untreated HIV patients, and the paired student t-tests across the paired patients’ samples at baseline and 12 months. GraphPad Prism 7 software was used in the rendering of graphs and figures.

Two types of immune-metabolic correlation networks were done. Firstly, for the control group and the untreated HIV patient group, independent Spearman correlation analyses were done using the absolute metabolite and cytokine levels. Next, per group cytokine levels were correlated with each other to form the network skeleton. The second type of correlation network was based on Spearman correlations of the levels of metabolites to cytokines in HIV-suppressed patients after 12 months of cART relative to the levels at baseline from paired patient samples (relative change = 12 months cART – baseline). Next, we correlated the relative change in cytokine levels to form the network skeleton. For both types of immune-metabolic correlations significant correlations were defined using a cut-off p-value (two-tailed) < 0.01 and were visualized as a network using the Metscape application within Cytoscape (v3.4.0).

### Ethics approval and consent to participate

This study was conducted in accordance with the guidelines of the Declaration of Helsinki and the principles of Good Clinical Practice. The ethical review board of the Erasmus MC has approved the study, and informed consent was obtained from all patients who were asked to donate blood.

## Electronic supplementary material

Supplementary data

## Data Availability

The metabolomics datasets supporting the conclusions of this article will be made available in the online MetaboLights data repository.
