# Supplementary material for: Persistent metabolic changes in HIV-infected patients during the first year of combination antiretroviral therapy
Source: Sci Rep. 2018 Nov 16;8:16947. doi: 10.1038/s41598-018-35271-0 (PMC6240055; doi:10.1038/s41598-018-35271-0)
Supplement: Supplementary file 1 — Supplementary data [file 41598_2018_35271_MOESM1_ESM.pdf]

Supplementary information

Persistent metabolic changes in HIV-infected patients during the first year of combination antiretroviral therapy

N. Chantal Peltenburg[1,8,ʃ], Johannes C. Schoeman[2,ʃ], Jun Hou[3], Fernando Mora[2], Amy C. Harms[2], Selwyn H. Lowe[4,5], Jörgen Bierau[6], Jaap A. Bakker[7], Annelies Verbon[1,8], Thomas Hankemeier[2], and Andre Boonstra[3*]

[1] Department of Internal Medicine, Division Infectious Diseases, Erasmus Medical Center, Wytemaweg 80, 3015 CE, Rotterdam, The Netherlands.

[2] Faculty of Science, Leiden Academic Centre for Drug Research, Analytical BioSciences, Einsteinweg 55, 2333 CC, Leiden, The Netherlands.

[3] Department of Gastroenterology and Hepatology, Erasmus Medical Center, Wytemaweg 80, 3015 CE, Rotterdam, The Netherlands.

[4] Department of Internal Medicine, Division Infectious Diseases, Maastricht University Medical Center, P. Debyelaan 25, 6229 HX, Maastricht, The Netherlands

[5] Department of Medical Microbiology, School of CAPHRI, Maastricht University Medical Center, P. Debyelaan 25, 6229 HX, Maastricht, The Netherlands

[6] Department of Clinical Genetics, Maastricht University Medical Center, P. Debyelaan 25, 6229 HX, Maastricht, The Netherlands

[7] Department of Clinical Chemistry and Laboratory Medicine, Leiden University Medical Center, Albinusdreef 2, 2333 ZA, Leiden, The Netherlands

[8] Department of Medical Microbiology and Infectious Diseases, Erasmus Medical Center, Wytemaweg 80, 3015 CE, Rotterdam, The Netherlands.

Supplementary Figures

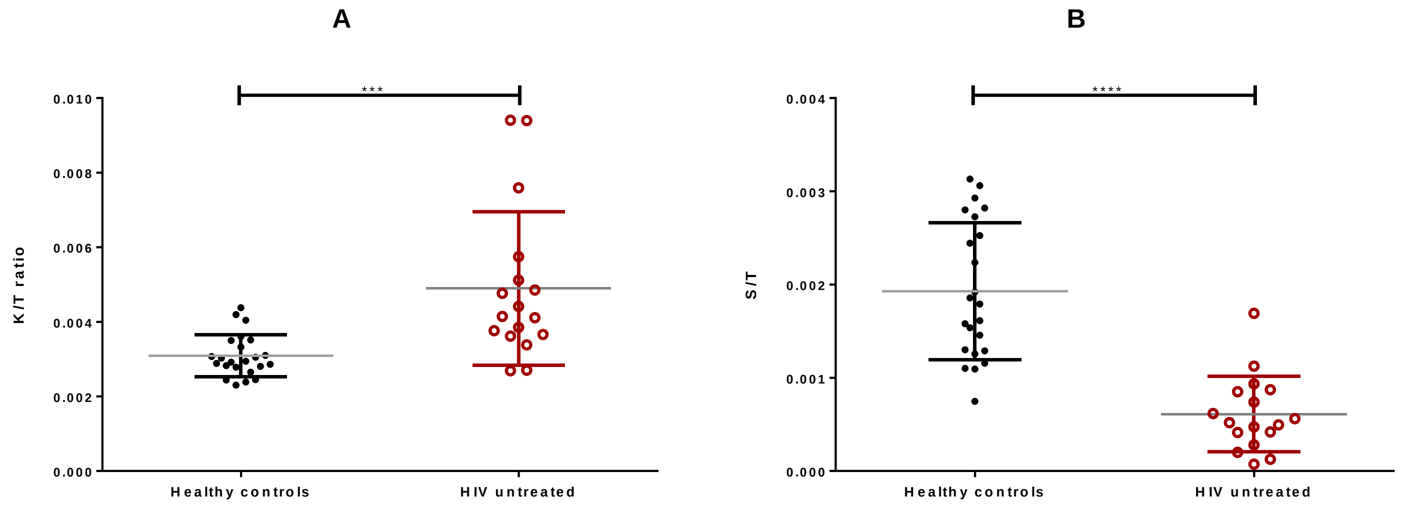

Fig. S1: **Tryptophan pathway and Indoleamine 2,3-dioxygenase (IDO) activity during an untreated HIV infection**. A - IDO activity during an untreated HIV infection (population A). The kynurenine and tryptophan (K/T) ratio representative of IDO activity are plotted for the controls (Black dots) and untreated HIV group (Red circles). B – The serotonin and tryptophan (S/T) ratio showing the altered tryptophan utilization during untreated HIV infection, with the controls (Black dots) and untreated HIV group (Red circles). Unpaired t-test, *** p< 0.001 with **** p< 0.0001.

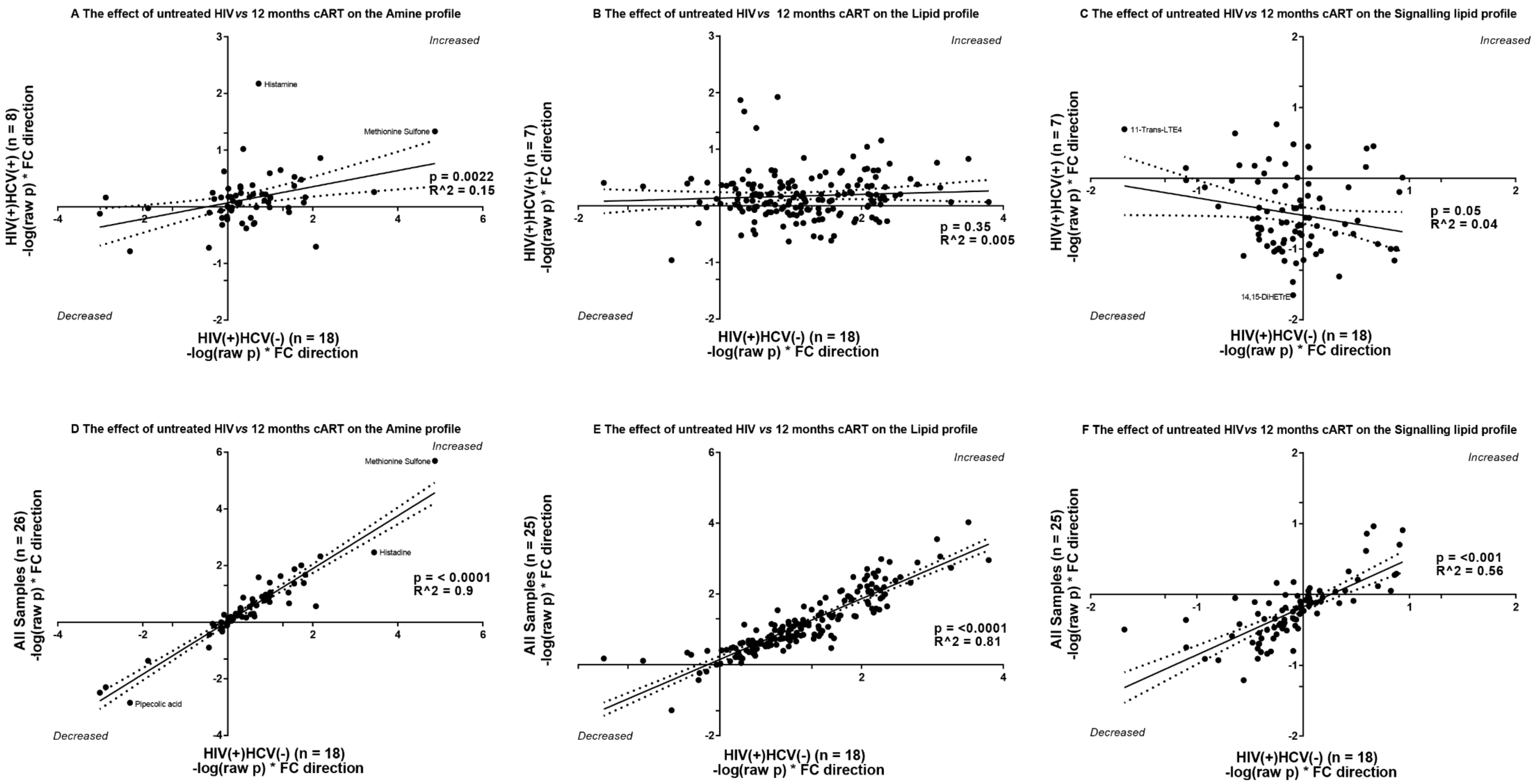

Fig. S2 Directed p-value plots investigating metabolic changes in paired baseline (0 months) and 12 months cART samples (population B), through comparing the metabolic responses in different patient sub-populations. The directed p-value plots shown in A, B, and C for the three different metabolomics platforms compares the HIV(+)HCV(-) and HIV(+)HCV(+) sub-populations. The directed p-value plots shown in D, E, and F for the three different metabolomics platforms compares the HIV(+)HCV(-) and HIV(+)HCV(+/-)(All samples) sub-populations. A significant degree of correlation is found in D, E and F revealing that the HCV(+) populations has little influence in skewing the data and in actual effect somewhat dampening the observed effect of 12 months cART. A paired student t-test was used, and the direction of change was determined by the fold change direction. Pearson correlations were done per platform to investigate the degree of correlation with R^2 and p-values reported per graph, dashed lines represent 95% confidence intervals.

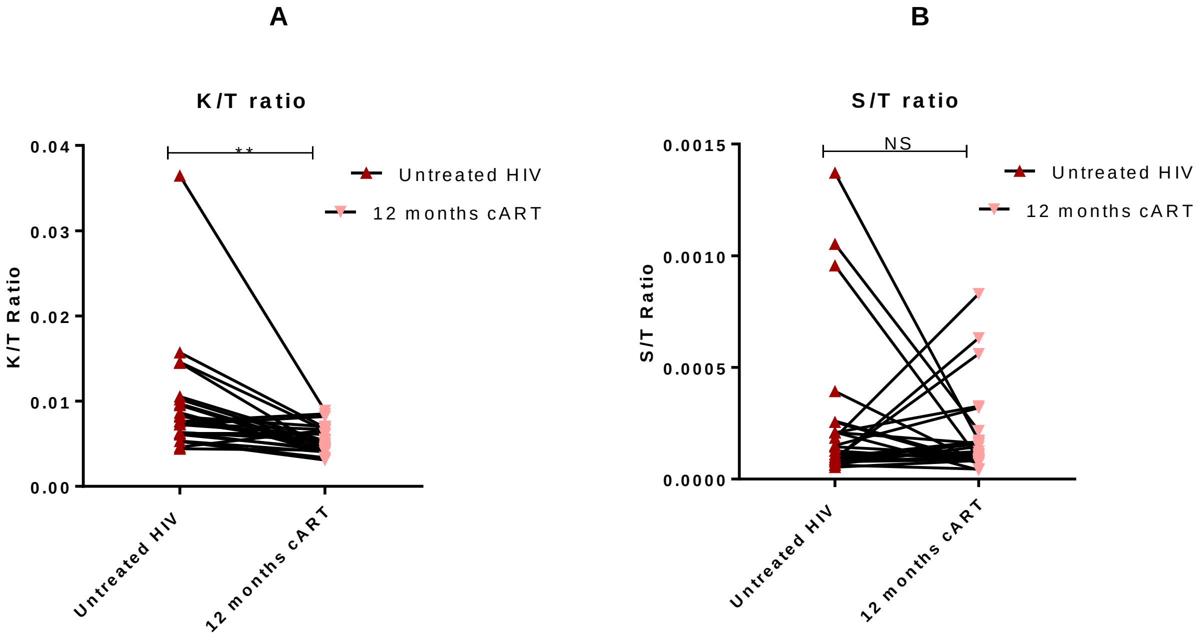

Fig. S3: **Tryptophan and Indoleamine 2,3-dioxygenase (IDO) activity during cART**. A - IDO activity decreased during 12 months of cART (population B). The kynurenine and tryptophan (K/T) ratio representative of IDO activity are plotted for the paired untreated HIV and 12 months cART samples. B – The serotonin and tryptophan (S/T) ratio showed nonsignificant changes during the first 12months of cART in the same patients. Paired t-test with ** p< 0.01.

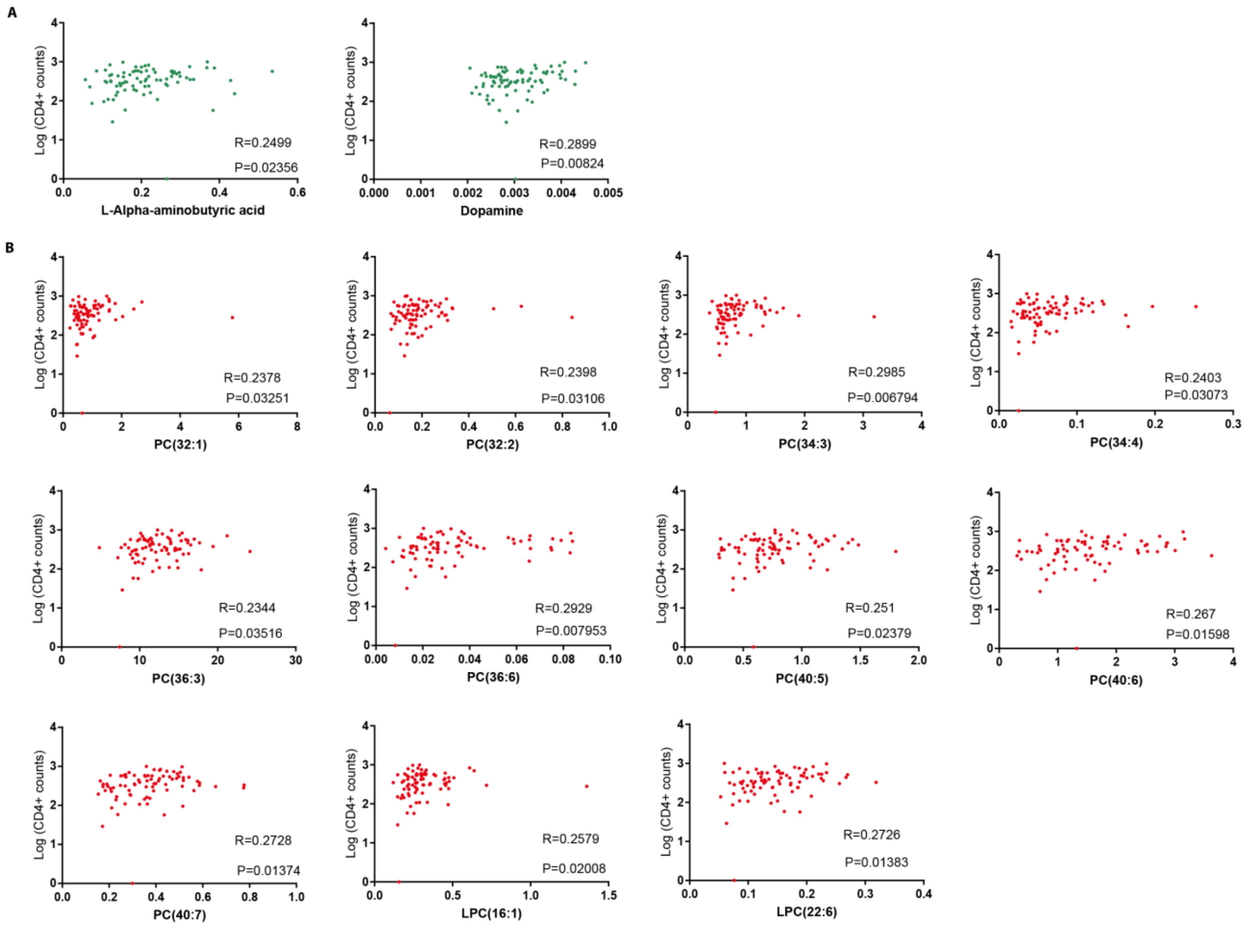

Fig. S4: Significant correlations between the CD4^{+} T-cell count and metabolite levels during 12 months of cART (population B). A – Amine (green) metabolites correlated significantly ($p<0.05$) to CD4^{+} T-cell counts. B – Phospholipid (red) metabolites correlated significantly ($p<0.05$) to CD4^{+} T-cell counts**.** All correlations were done using Spearman.

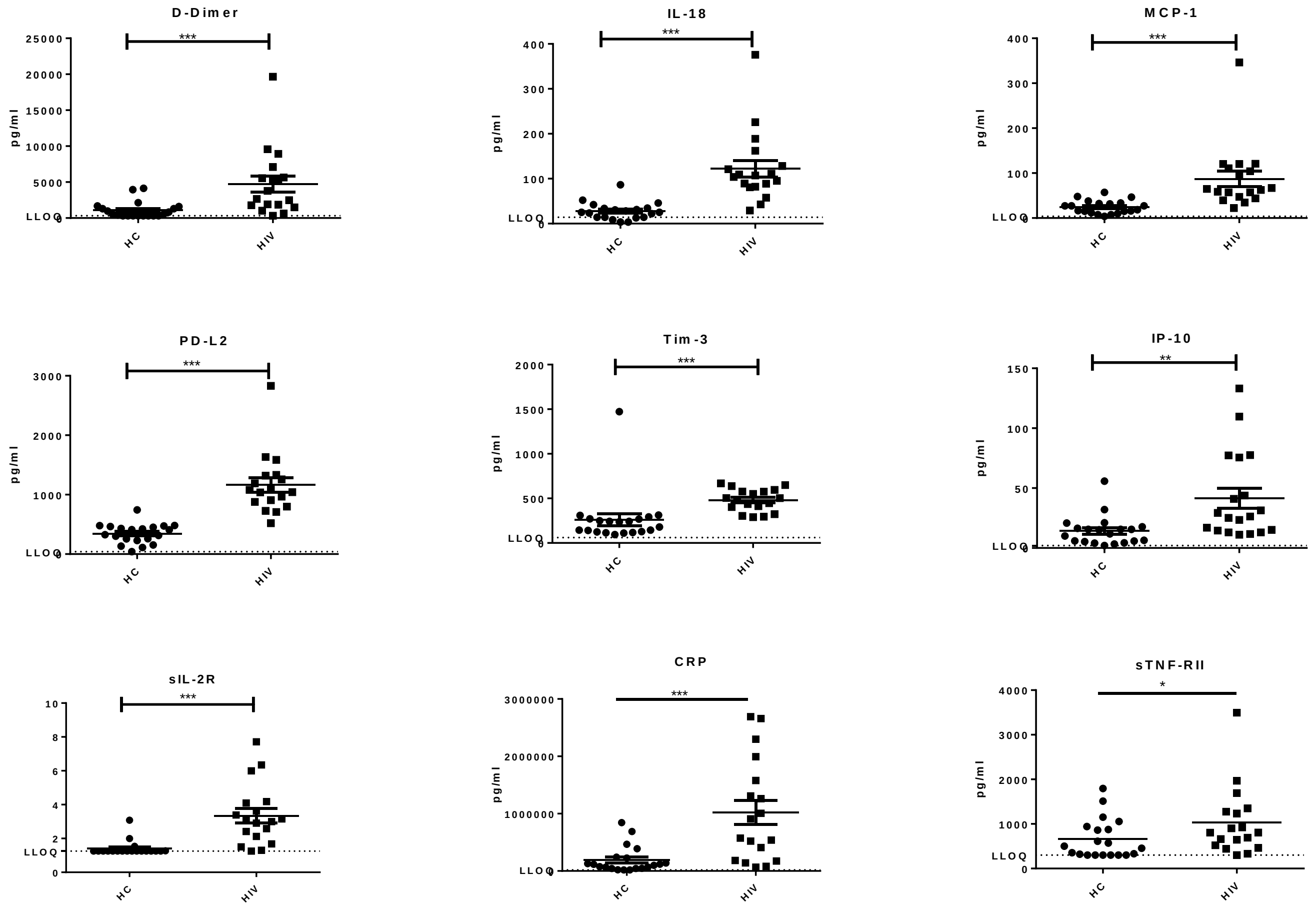

Fig. S5: Plasma levels of the detected immunological markers in untreated HIV patients vs. controls of population A. Increased levels of all markers were found during an untreated HIV infection. Unpaired t-test with p-values: * <0.05, ** <0.01, and *** <0.001.

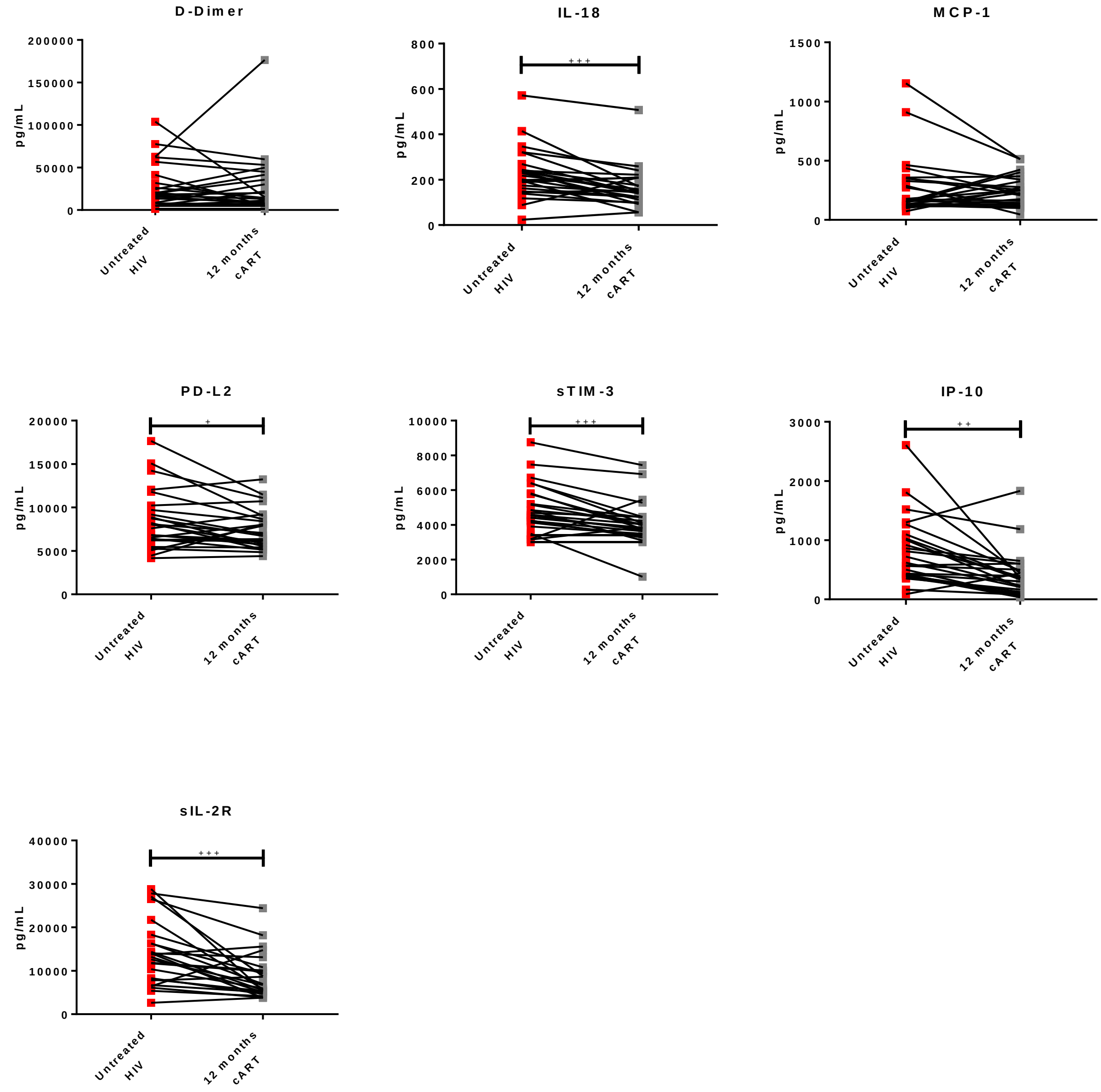

Fig. S6: Plasma levels of the detected immunological markers in paired patient samples of population B. Decreasing levels of most markers were found during the first 12 months of cART. Paired t-test with p-values: + < 0.05, ++ < 0.01, +++ <0.001.

Supplementary Tables

Table S1: Descriptive statistics of the biogenic amines comparing the untreated HIV patients to the controls in patient population A.

Metabolite	T-test	False discovery rate	Fold Change	Controls	Untreated HIV
	p-value	q-value	(FC)	Mean ± SD	Mean ± SD
Glutathione	1.22E-11	3.88E-10	0.43017	0.12 ± 0.03	0.050 ± 0.01
O-Phosphoethanolamine	1.38E-11	3.88E-10	0.38745	3.71 ± 1.20	1.44 ± 0.53
Sarcosine	1.72E-10	3.21E-09	0.53698	0.13 ± 0.03	0.071 ± 0.02
Serotonin	4.05E-10	5.67E-09	0.24411	0.006 ± 0.00	0.002 ± 0.00
Taurine	6.14E-08	6.88E-07	0.50741	0.66 ± 0.26	0.33 ± 0.11
L-Tryptophan	1.59E-05	0.000149	0.76073	3.25 ± 0.51	2.47 ± 0.46
L-Alanine	0.000179	0.001432	0.76731	3.88 ± 0.79	2.98 ± 0.54
L-Histidine *	0.000396	0.002773	0.81706	0.037 ± 0.01	0.030 ± 0.01
L-Asparagine	0.000463	0.002882	0.77301	0.80 ± 0.18	0.62 ± 0.11
L-Alpha-aminobutyric acid	0.000848	0.004749	0.6884	0.27 ± 0.09	0.19 ± 0.04
L-Carnosine	0.000948	0.004826	0.63414	0.000 ± 0.00	0.000 ± 0.00
Ethanolamine *	0.002506	0.011693	0.85843	0.27 ± 0.03	0.23 ± 0.05
L-Leucine	0.002953	0.012719	0.7675	1.36 ± 0.36	1.04 ± 0.22
Gamma-L-glutamyl-L-alanine	0.004387	0.017546	0.73374	0.008 ± 0.00	0.006 ± 0.00
L-Methionine	0.005782	0.020692	0.75999	1.43 ± 0.45	1.09 ± 0.24
Putrescine	0.005912	0.020692	0.64703	0.001 ± 0.00	0.001 ± 0.00
L-Tyrosine	0.006596	0.021727	0.76809	9.06 ± 2.63	6.96 ± 1.61
L-Valine *	0.011206	0.034862	0.82117	7.46 ± 1.68	6.12 ± 1.20
Ornithine #	0.017497	0.051569	0.79523	1.63 ± 0.49	1.29 ± 0.37
S-Methylcysteine #	0.023788	0.066607	0.65039	0.13 ± 0.09	0.083 ± 0.03
L-Isoleucine #	0.025046	0.066789	0.78844	3.62 ± 1.16	2.86 ± 0.69
Gamma-Glutamylglutamine *#	0.028906	0.07358	0.8311	0.007 ± 0.00	0.006 ± 0.00
L-Lysine *#	0.035184	0.085666	0.83576	2.04 ± 0.55	1.71 ± 0.33

* Metabolites adhering to $p < 0.05$ but not a FC of 30 % (FC > 1.3 or FC < 0.7)
\# Metabolites having a FDR q-value > 0.05

Table S2: Descriptive statistics of the lipid profiling comparing the untreated HIV patients to the controls in patient population A.

Metabolite	T-test	False discovery rate	Fold Change	Controls	Untreated HIV
	p-value	q-value	(FC)	Mean ± SD	Mean ± SD
CE205 #	0.0250	0.123	0.774	0.03 ± 0.01	0.02 ± 0.01
CE226 #	0.0106	0.073	0.808	0.034 ± 0.01	0.027 ± 0.01
Cerd180_220 #	0.0451	0.179	0.769	0.033 ± 0.01	0.025 ± 0.01
Cerd180_240 #	0.0226	0.118	0.685	0.041 ± 0.02	0.028 ± 0.01
Cerd181_160 #	0.0114	0.073	1.490	0.01 ± 0.00	0.02 ± 0.01
LPA-160	0.0003	0.002	2.005	0.09 ± 0.04	0.18 ± 0.13
LPA-161	0.0021	0.013	1.992	0.01 ± 0.01	0.03 ± 0.02
LPA-181	0.0017	0.011	1.847	0.10 ± 0.04	0.19 ± 0.15
LPA-182 #	0.0256	0.083	1.515	0.56 ± 0.19	0.85 ± 0.57
LPA-203	0.0003	0.002	2.165	0.02 ± 0.01	0.04 ± 0.02
LPA-204	0.0000	0.000	2.047	0.15 ± 0.05	0.30 ± 0.16
LPC182 #	0.0104	0.073	0.753	3.97 ± 1.30	2.99 ± 0.97
PC322 #	0.0449	0.179	0.734	0.22 ± 0.09	0.16 ± 0.08
PC342 *#	0.0109	0.073	0.829	24.63 ± 5.54	20.42 ± 3.54
PC362 *	0.0017	0.028	0.837	18.11 ± 2.80	15.16 ± 3.02
PC365 #	0.0405	0.169	0.657	1.25 ± 0.73	0.82 ± 0.52
PC366 #	0.0103	0.073	0.661	0.04 ± 0.02	0.02 ± 0.01
PC386	0.0003	0.012	0.702	5.56 ± 1.68	3.90 ± 1.17
PC404	0.0007	0.016	1.323	0.12 ± 0.04	0.16 ± 0.03
PC406	0.0014	0.026	0.712	1.64 ± 0.54	1.17 ± 0.45
PC407	0.0001	0.008	0.685	0.35 ± 0.10	0.24 ± 0.07
PC408	0.0001	0.008	0.653	0.08 ± 0.03	0.05 ± 0.02
PCO-341 *#	0.0081	0.072	0.833	0.40 ± 0.08	0.33 ± 0.08
PCO-342	0.0001	0.008	0.707	0.54 ± 0.13	0.38 ± 0.10
PCO-343	0.0008	0.016	0.690	0.52 ± 0.16	0.36 ± 0.13
PCO-363	0.0003	0.012	0.747	0.13 ± 0.03	0.10 ± 0.02
PCO-386 #	0.0143	0.085	0.779	0.06 ± 0.04	0.05 ± 0.03
PCO-387 *#	0.0162	0.090	0.813	0.10 ± 0.02	0.08 ± 0.03
PE342 #	0.0386	0.169	0.705	0.41 ± 0.20	0.29 ± 0.09
PE382	0.0045	0.050	0.703	3.13 ± 1.22	2.20 ± 0.70
PEO-385 #	0.0200	0.108	0.777	0.67 ± 0.24	0.52 ± 0.18
PEO-387	0.0004	0.012	0.636	0.24 ± 0.09	0.16 ± 0.05
SMd181_180 *#	0.0406	0.169	0.839	1.00 ± 0.25	0.84 ± 0.22
SMd181_181 #	0.0243	0.123	0.796	0.40 ± 0.12	0.32 ± 0.11
SMd181_201	0.0019	0.028	0.750	0.28 ± 0.08	0.21 ± 0.06
TG501 #	0.0156	0.090	1.346	7.59 ± 4.97	10.22 ± 4.24
TG502 #	0.0099	0.073	1.360	10.73 ± 5.05	14.59 ± 5.60
TG503 #	0.0364	0.169	1.359	5.54 ± 2.88	7.52 ± 3.54
TG511 #	0.0114	0.073	1.526	0.57 ± 0.51	0.86 ± 0.45
TG512 #	0.0075	0.069	1.589	0.97 ± 0.64	1.55 ± 0.82
TG513 #	0.0067	0.066	1.414	0.70 ± 0.28	0.99 ± 0.38
TG514 #	0.0469	0.182	1.281	0.30 ± 0.12	0.38 ± 0.14
TG522 #	0.0125	0.077	1.296	30.19 ± 7.26	39.13 ± 12.24
TG531 #	0.0393	0.169	1.375	0.13 ± 0.08	0.18 ± 0.08
TG552 #	0.0277	0.132	1.284	0.09 ± 0.03	0.12 ± 0.04
TG565	0.0036	0.043	1.593	2.00 ± 0.52	3.19 ± 1.54

TG566 #	0.0052	0.054	1.573	2.73	±	0.74	4.29	±	1.96
TG585	0.0034	0.043	1.535	0.14	±	0.04	0.22	±	0.12
TG586	0.0020	0.028	1.514	0.28	±	0.06	0.42	±	0.19

* Metabolites adhering to p< 0.05 but not a FC of 30 % (FC > 1.3 or FC < 0.7)
[#] Metabolites having a FDR q-value > 0.05

Table S3: Descriptive statistics of the signalling lipid profiling. Comparing the untreated HIV patients to the controls in patient population A.

Metabolite	T-test	False discovery rate	Fold Change	Controls			Untreated HIV		
	p-value	q-value	(FC)	Mean ± SD			Mean ± SD		
PGF2a *#	0.01302	0.0509	1.1522	0.029	±	0.004	0.033	±	0.0065
PGE2 #	0.03223	0.0904	2.1036	0.015	±	0.005	0.028	±	0.0255
12S-HHTrE #	0.03437	0.0943	1.4853	0.460	±	1.018	0.684	±	0.6429
5,6-DiHETrE	0.00000	0.0000	2.1354	0.006	±	0.003	0.013	±	0.0060
14,15-DiHETrE	0.00000	0.0000	1.5507	0.047	±	0.009	0.073	±	0.0212
11,12-DiHETrE	0.00000	0.0001	1.5794	0.036	±	0.008	0.057	±	0.0183
8,9-DiHETrE	0.00001	0.0002	1.644	0.006	±	0.002	0.010	±	0.0033
5-HETE	0.00007	0.0008	1.8677	0.061	±	0.024	0.114	±	0.0457
15-HETE	0.00079	0.0060	1.6121	0.088	±	0.040	0.143	±	0.0593
15S-HETrE	0.00256	0.0138	1.6857	0.037	±	0.023	0.062	±	0.0319
12-HETE	0.00566	0.0252	2.265	0.779	±	0.298	1.765	±	1.1447
12S-HEPE #	0.01717	0.0633	0.61502	0.263	±	0.176	0.161	±	0.1160
9/10-NO2-OA	0.00058	0.0047	0.49727	0.145	±	0.117	0.072	±	0.0477
11-HDoHE	0.00000	0.0000	0.25878	0.172	±	0.074	0.045	±	0.0180
2,3-dinor-8-iso-PGF2a	0.00000	0.0001	2.2174	0.086	±	0.027	0.191	±	0.0817
20-HETE	0.00223	0.0131	1.5512	0.071	±	0.020	0.110	±	0.0497
8,12-iPF2a-IV	0.00250	0.0138	0.80572	0.032	±	0.006	0.025	±	0.0087
8-iso-PGF2a	0.00533	0.0245	0.74017	0.133	±	0.039	0.098	±	0.0384
8-iso-PGE2 #	0.04270	0.1102	2.6156	0.009	±	0.006	0.024	±	0.0188
SPHA-C180	0.00394	0.0195	1.6675	0.074	±	0.023	0.123	±	0.0825
S-1-P-161	0.00454	0.0217	0.7423	0.220	±	0.069	0.163	±	0.0450
S-1-P-182	0.00746	0.0321	0.77115	0.951	±	0.250	0.734	±	0.2526
S-1-P-181 #	0.02076	0.0724	0.79819	2.914	±	0.783	2.326	±	0.7220

* Metabolites adhering to p< 0.05 but not a FC of 30 % (FC > 1.3 or FC < 0.7)
# Metabolites having a FDR q-value > 0.05

Table S4: Descriptive statistics of the biogenic Amine and lipid profiling. Comparing the untreated HIV patients to their paired 12 months cART follow samples in patient population B.

Platform	Metabolite	Paired T-test	False discovery rate	T-stat[#]	Untreated HIV (Baseline)			12 months cART		
		p-value	q-value		Mean	±	SD	Mean	±	SD
Biogenic amines	Methionine sulfone	2.0E-06	1E-04	6.14	0.008	±	0.003	0.014	±	0.007
	L-Pipecolic acid	0.0014	3E-02	-3.59	0.046	±	0.015	0.040	±	0.016
	DL-3-aminoisobutyric acid	0.0032	4E-02	-3.26	0.023	±	0.011	0.018	±	0.008
	L-Histidine	0.0034	4E-02	3.23	0.022	±	0.003	0.025	±	0.004
	L-Tryptophan	0.0047	5E-02	3.10	2.715	±	0.891	3.243	±	0.487
	L-Kynurenine	0.0051	5E-02	-3.07	0.021	±	0.006	0.017	±	0.004
Lipid profiling	CE181	0.0086	5E-02	2.86	0.039	±	0.011	0.047	±	0.009
	LPC181	0.0079	5E-02	2.90	2.372	±	0.681	2.751	±	0.558
	LPC183	0.0030	4E-02	3.30	0.054	±	0.020	0.077	±	0.029
	LPC203	0.0065	5E-02	2.98	0.291	±	0.103	0.369	±	0.116
	PC322*	0.0127	6E-02	2.69	0.149	±	0.061	0.192	±	0.068
	PC341	0.0011	3E-02	3.71	12.278	±	2.382	14.096	±	2.540
	PC343	0.0009	3E-02	3.80	0.658	±	0.195	0.886	±	0.269
	PC344 *	0.0115	6E-02	2.74	0.047	±	0.031	0.070	±	0.039
	PC361	0.0003	2E-02	4.25	2.712	±	0.891	3.663	±	1.115
	PC362	0.0012	3E-02	3.66	15.778	±	3.018	18.441	±	3.238
	PC363	0.0018	4E-02	3.51	10.626	±	2.977	13.234	±	2.799
	PC364 *	0.0121	6E-02	2.71	11.041	±	2.766	12.950	±	2.812
	PC382	0.0013	3E-02	3.64	0.322	±	0.102	0.432	±	0.124
	PC383*	0.0165	7E-02	2.58	2.514	±	1.250	3.699	±	1.838
	PC384*	0.0141	6E-02	2.65	7.179	±	2.238	9.006	±	2.590
	PC385*	0.0141	6E-02	2.65	3.437	±	1.338	4.544	±	1.349
	PC404*	0.0102	5E-02	2.79	0.169	±	0.065	0.223	±	0.083
	PC405	0.0052	4E-02	3.07	0.605	±	0.200	0.876	±	0.292
	PC406*	0.0226	8E-02	2.44	1.231	±	0.415	1.821	±	0.866
	PC407	0.0083	5E-02	2.88	0.311	±	0.115	0.418	±	0.146
	PC408	0.0033	4E-02	3.27	0.058	±	0.022	0.077	±	0.024
	PCO-341	0.0039	4E-02	3.20	0.358	±	0.068	0.398	±	0.075
	PCO-342	0.0001	2E-02	4.68	0.532	±	0.123	0.669	±	0.140
	PCO-343	0.0033	4E-02	3.26	0.391	±	0.129	0.466	±	0.101
	PCO-362	0.0026	4E-02	3.36	0.188	±	0.033	0.212	±	0.035
	PCO-363	0.0022	4E-02	3.43	0.128	±	0.028	0.152	±	0.028
	PCO-385	0.0048	4E-02	3.11	0.861	±	0.162	0.986	±	0.192
	PCO-406	0.0062	5E-02	3.00	0.143	±	0.021	0.169	±	0.029
	PE382	0.0010	3E-02	3.73	3.248	±	0.995	4.343	±	1.346
	PE384*	0.0180	7E-02	2.54	0.203	±	0.073	0.286	±	0.146
	PEO-365	0.0042	4E-02	3.16	0.300	±	0.109	0.370	±	0.144
	PEO-385	0.0079	5E-02	2.90	0.460	±	0.169	0.580	±	0.243
	SMd181/161*	0.0276	9E-02	2.35	1.033	±	0.275	1.161	±	0.248
	SMd181/181*	0.0289	1E-01	2.32	0.706	±	0.225	0.821	±	0.244
	SMd181/200	0.0033	4E-02	3.26	1.210	±	0.274	1.470	±	0.351
	SMd181/201	0.0020	4E-02	3.47	0.403	±	0.112	0.490	±	0.142
	SMd181/210*	0.0123	6E-02	2.71	0.367	±	0.107	0.441	±	0.128
	SMd181/220	0.0067	5E-02	2.97	2.761	±	0.647	3.173	±	0.649
	SMd181/221	0.0037	4E-02	3.21	1.866	±	0.395	2.159	±	0.393
	SMd181/230	0.0084	5E-02	2.87	1.028	±	0.250	1.176	±	0.267
	SMd181/231	0.0060	5E-02	3.01	0.758	±	0.137	0.872	±	0.170
	SMd181/240*	0.0099	5E-02	2.80	1.827	±	0.457	2.090	±	0.431
	SMd181/241*	0.0135	6E-02	2.67	6.005	±	1.019	6.506	±	1.023
	SMd181/242	0.0084	5E-02	2.87	2.506	±	0.575	2.751	±	0.529
	TG505*	0.0155	6E-02	2.61	0.178	±	0.232	0.248	±	0.223
	TG525*	0.0257	9E-02	2.38	2.160	±	2.715	2.727	±	1.860
	TG545*	0.0278	9E-02	2.34	7.446	±	6.853	9.387	±	5.391
	TG546*	0.0223	8E-02	2.44	2.985	±	3.473	3.687	±	2.546
	TG547*	0.0261	9E-02	2.37	0.688	±	0.895	0.844	±	0.650
	TG563*	0.0192	7E-02	2.51	0.596	±	0.582	0.756	±	0.489

* Metabolites adhering to p< 0.05 but not a FDR q-value of q< 0.05
# For t.stat positive values = increased in 12 months vs Untreated. Negative values = decrease in 12 months vs Untreated
